# TRPV1 channel-mediated bilateral allodynia induced by unilateral masseter muscle inflammation in rats

**DOI:** 10.1186/1744-8069-9-68

**Published:** 2013-12-30

**Authors:** Suncana Simonic-Kocijan, Xuehong Zhao, Wen Liu, Yuwei Wu, Ivone Uhac, KeWei Wang

**Affiliations:** 1 Department of Neurobiology, Neuroscience Research Institute, Peking University Health Science Center, Beijing, China; 2 Department of Prosthodontics, School of Dental Medicine, Medical Faculty, Rijeka University, Krešimirova 40, 51000, Rijeka, Croatia; 3 Medical function Department, Medical College of Hubei University of Arts and Science, Xiangyang 441053, China; 4 The 2nd Dental Center, Peking University School of Stomatology, 38 Xueyuan Road, Beijing 100191, China; 5 Department of Molecular and Cellular Pharmacology, Peking University School of Pharmaceutical Sciences, 38 Xueyuan Road, Beijing 100191, China; 6 PKU-IDG/McGovern Institute for Brain Research, Peking University, Beijing 100871, China

**Keywords:** Hippocampus, Masseter muscle, TMD, TRG, TRPV1

## Abstract

Pain in masticatory muscles is among the most prominent symptoms of temperomandibular disorders (TMDs) that have diverse and complex etiology. A common complaint of TMD is that unilateral pain of craniofacial muscle can cause a widespread of bilateral pain sensation, although the underlying mechanism remains unknown. To investigate whether unilateral inflammation of masseter muscle can cause a bilateral allodynia, we generated masseter muscle inflammation induced by unilateral injection of complete Freund’s adjuvant (CFA) in rats, and measured the bilateral head withdrawal threshold at different time points using a von Frey anesthesiometer. After behavioral assessment, both right and left trigeminal ganglia (TRG) were dissected and examined for histopathology and transient receptor potential vanilloid 1 (TRPV1) mRNA expression using quantitative real-time PCR analysis. A significant increase in TRPV1 mRNA expression occurred in TRG ipsilateral to CFA injected masseter muscle, whereas no significant alteration in TRPV1 occurred in the contralateral TRG. Interestingly, central injection of TRPV1 antagonist 5-iodoresiniferatoxin into the hippocampus significantly attenuated the head withdrawal response of both CFA injected and non-CFA injected contralateral masseter muscle. Our findings show that unilateral inflammation of masseter muscle is capable of inducing bilateral allodynia in rats. Upregulation of TRPV1 at the TRG level is due to nociception caused by inflammation, whereas contralateral nocifensive behavior in masticatory muscle nociception is likely mediated by central TRPV1, pointing to the involvement of altered information processing in higher centers.

## Introduction

Temporomandibular disorders (TMDs) are the most common orofacial pain conditions affecting musculoskeletal and joint tissue [1]. TMD affects 4-12% of population with masticatory muscles pain as the most prominent patient complaint [2-4]. It is estimated that masticatory myalgia characterized with pain and tenderness covers half of overall TMD [5]. Despite the fact that masticatory muscle pain is very common the underlying mechanism is still not well understood. Evidence of inflammation in myogenous TMD is not certain, although some previous studies report the muscle tissue damage and inflammation induced by parafunctional habits, and an association between release of inflammatory mediators and pain of masticatory muscles [6,7]. It seems that patients with TMD can exhibit altered central nociceptive processing, which is thought to be triggered from a peripheral source possibly from masticatory muscles [8,9], as nociceptive inputs from inflammatory muscles are a potent generator of CNS wind-up that starts at the skin and culminates in hypersensitivity response from the dorsal horn and brain [10]. The involvement of neural mechanisms in etiopathogenesis of TMD is supported by the fact that unilateral inflammation of the masseter muscle in rats and muscle pain in humans is able to induce a bilateral and widespread mechanical allodynia and hyperalgesia [11-14]. Accumulating evidence suggests a role of various ion channels at both peripheral and central level in nociception of deep craniofacial tissues [15-17]. For instance, recent studies demonstrate that transient receptor potential vanilloid 1 (TRPV1) plays an important role in orofacial pain sensitivity in animal models following inflammation of the temporomandibular joint (TMJ) and masticatory muscles [16,18], and central activation of voltage-gated Kv7/KCNQ/M potassium channels attenuates hyperalgesia induced by TMJ inflammation [17].

TRPV1 plays a key role in peripheral inflammatory and neuropathic pain states [19]. They are expressed in the sensory nerves of both peripheral and central nervous system, including masseter muscle afferents in the trigeminal ganglion (TRG) neurons [20] and hippocampus [16,21]. Although the role of TRPV1 in craniofacial muscle pain is not certain, studies suggest that changes in TRPV1 expression may contribute to orofacial pain sensitivity. Peripheral application of capsaicin induces sensitization in trigeminal nociceptive afferents innervating deep craniofacial tissues [22,23]. The fact that intramuscular injection with specific TRPV1 agonist produces prolonged mechanical hyperalgesia suggests that TRPV1 channel expression in muscle afferents contributes to the development of pathologic muscle pain conditions [24]. Findings of contralateral and referred pain in humans after muscle and skin capsaicin injection [25,26] and the dependency of contralateral pain on TRPV1 [27] after unilateral paw inflammation implicate the involvement of TRPV1 in bilateral hyperalgesia.

Although the hippocampus is not recognized as a major area in the brain involved in pain processing, there is possible hippocampal involvement in various pain conditions because painful stimuli can induce changes of neuronal activity, blood flow, and c-fos expressions in the hippocampus [28-30]. Hippocampal TRPV1 can directly mediate synaptic plasticity and is involved in anxiety-related behaviors and conditioned fear [31-33], implying that hippocampal TRPV1 could be related to the affective or cognitive aspects of pain. Recent findings of changes at hippocampal level in fibromyalgia and low back pain conditions support the possibility of hippocampal involvement in various pain states [34,35]. Findings of upregulation of TRPV1 in the hippocampus but not in other brain regions after muscle inflammation suggest a role for hippocampal TRPV1 in TMD, pointing to the importance of hippocampus in central pain processing from orofacial region [16].

Despite growing interest in understanding masticatory muscle pain conditions the pathophysiological mechanisms leading to the development of masticatory muscle pain are still poorly understood. It is not clear whether unilateral inflammation can induce bilateral nocifensive response, and if so, whether the changes in nociception are regulated by TRPV1 channel function. The aim of this study was to investigate the nocifensive behavioral response and changes in TRPV1 channel expression in TRG neurons both at the site of inflammation and at contralateral side following unilateral injection of CFA into the masseter muscle. Our findings show that the involvement of central TRPV1 in bilateral allodynia induced by unilateral masseter muscle inflammation.

## Materials and methods

### Animals

Adult male Sprague Dawley rats (250–300 g) with total of 46 were used in this study. Rats were housed in groups (two to five per cage) under a controlled temperature (22 ± 1°C) and humidity (50 ± 5%) environment with ad libitum access to food and water. Animals were maintained on a 12 h/12 h light/dark cycle. The animal experimental protocols were approved by the Animal Use and Care Committee of Peking University and were consistent with the Ethical Guidelines of the International association for the Study of Pain.

### Induction of masseter muscle inflammation in rats

Each animal was anesthetized with sodium pentobarbital (50 mg/kg i.p.), and injected with 50 μL complete Freund’s adjuvant (CFA, Sigma F5881, 0.5 mg/mL, heat killed *Mycobacterium tuberculosis* suspended in oil: saline at 1:1 emulsion) into the mid-region of right masseter muscle (n = 6), as described in previous studies [11,36]. For the control rats, 50 μL of 0.9% saline was injected into the right masseter muscle (n = 6). To ensure that either CFA or saline was administered in the same region, the injection site was determined by palpating the masseter muscle between the angle of the mandible and the zygomatic bone. All injections were made using a 27-gauge needle. Upon contacting the mandible the needle was slowly withdrawn into the mid-region of the masseter and injections were completed within 5–10 seconds [24]. Animals were closely monitored for evidence of edema after injection of CFA. Development of masseter muscle inflammation was examined by histopathology.

### Evaluation of masseter muscle allodynia

To evaluate nocifensive behavioral response after unilateral CFA or saline injection into masseter muscle bilateral mechanical head withdrawal threshold was measured at different time points using a von Frey anesthesiometer (IITC Life Science) with the tip size of 1.0 mm as previously reported in investigations of deep tissue inflammatory pain conditions [37,38]. Initially, animals were habituated to stand unrestrained on their hind paws and lean on the experimenter’s hand enclosed in a leather glove. Mechanical thresholds were then tested by probing bilateral masseter muscles at time points of 4 hours, day 1, day 4, day 7 nd day 11 in the groups of either CFA or saline injection. The force needed to elicit a withdrawal of the head was recorded following five-stimulus presentations at approximately one minute interval, and average of these five measurements was used as the withdrawal threshold. Behavioral assessment procedures were conducted on the basis of double-blind design where the investigator who was blind to drug administration and grouping conducted behavioral measurements.

### Tissue preparation and histopathological examination

Four days after CFA (n = 8) or saline injection (n = 8), head withdrawal threshold of the additional rats was measured. After behavioral assessment, rats were decapitated and both right and left trigeminal ganglia from CFA rats and control animals were dissected for RNA extraction, frozen in liquid nitrogen and stored at −80°C until processed. Bilateral masseter muscles were fixed in 4% paraformaldehide, embedded in paraffin, cut at 40 μm and stained with haematoxylin and eosin.

### Quantitative real-time PCR

Total RNA was extracted from trigeminal ganglion (TRG) with TRIzol and was digested with 6 μl Turbo DNase for 1 hour. cDNA was synthesized using oligo dT and M-MLV reverse transcriptase according to the manufacturer’s instructions (Invitrogen). Real-time PCR amplification mixture (20 μl) contained 1 μl template cDNA, 10 μl 2× SYBR® Premix Ex TaqTM (Takara) containing SYBR Green and 5 μM forward and reverse primers. The mRNA level for gene of interest was acquired from the value of threshold cycle (Ct) as a relative level of β-actin through the formula 2-ΔCt (ΔCt = β-actin Ct - gene of interest Ct). The sequences of these primers (for β-actin, forward 5′-TGTTACCAACTGGGACGAC and reverse 5′- GGTGTTGAAGGTCTCAAACAT; for TRPV1, forward 5′-GACATGCCACCCAGCAGG and reverse 5′-TCAATTCCCACACACCTCCC) were designed using Oligo software [16]. The product of the TRPV1 primers used to amplify the TRPV1 gene was 261 bp. β-actin was used as an internal reference and the product length was 163 bp. Amplification was carried out using the ABI PRISM 7500™ with cycling conditions as follows: there was an initial denaturation step at 95°C for 10 s, followed by 40 cycles at 95°C for 5 s and 60°C for 34 s with fluorescent detection at 60°C. Melting curve analysis was performed from 60°C to 95°C in 1°C step. Results were analyzed using the 2^-ΔΔCt^ method to compare expression of genes of interest with that of β-actin.

### Intrahippocampal injection of TRPV1 antagonists

To test the effect of hippoampal TRPV1 on bilateral nocifensive behavioral response after unilateral masseter muscle inflammation, rats were anesthetized with sodium pentobarbital (50 mg/kg i.p., n = 18), and injected with 5-iodoresiniferatoxin (LC Laboratories, 0.1 or 0.5 nmol in 1 μl, dissolved in dimethylsulfoxide (DMSO) or vehicle (saline/DMSO at a ratio of 1:1) bilaterally into the CA1 region of hippocampus, according to our previous experimental procedures [16]. All solutions were freshly prepared before use, and the dose and volume of 5-iodo-resiniferatoxin were based on previous study [16]. Briefly, the anesthetized rat was placed in a stereotaxic frame, and two guide cannulas (5 mm in length, 0.6 mm of outer diameter, and 0.3 mm of inner diameter) were bilaterally inserted 1 mm above the CA1 region of the hippocampi (coordinates: -3.5 mm anteroposterior, -2.4 mm left to right (10° angle), and ± 2 mm dorsoventral relative to the bregma and skull surface). The cannulas were attached to the skull with self-curing acrylic resin cement. Rats were allowed to recover from surgery for at least 7 days before intrahippocampal injections of TRPV1 antagonist or vehicle was performed. Intrahippocampal injections of TRPV1 antagonists or vehicle into the bilateral CA1 regions of the hippocampi were performed with a micromanipulator mounted with two 1 μl-Hamilton microsyringes. Each microsyringe was connected through a PE-10 polyethylene catheter to an injection cannula that was introduced into the guide cannula with its tip extended 1.0 mm beyond the tip of the guide cannula. The bilateral hippocampi were simultaneously injected with 1 μl of TRPV1 antagonists or vehicle over 1 min, and the injection cannula was kept in place for an additional 1 min to allow for sufficient diffusion.

The effect of intrahippocampal injection of TRPV1 antagonist and vehicle on the baseline of head withdrawal threshold was initially examined. The head withdrawal threshold was measured for a period of 60 min after hippocampal injections. To examine the effect of intrahippocampal injection of TRPV1 antagonist on bilateral nocifensive response after unilateral masseter inflammation, all rats were injected with CFA into unilateral masseter muscle. Four days after CFA injection, bilateral head withdrawal threshold was measured to reconfirm development of bilateral mechanical allodynia. After behavioral assessment each rat was intrahippocampally injected with TRPV1 antagonist or vehicle, and head withdrawal thresholds were re-measured every 15 min for a period of 60 min. Rats were then decapitated and coronal sections (30 μm) of the brains were sliced on a cryostat, and the sections were examined under a microscope to confirm the placement of cannulas.

### Statistical analysis

GraphPad Prism 4.0 software (Graph-Pad Software, San Diego, CA) was used for all statistical analysis. All data are presented as mean ± SEM. Two-way repeated measures ANOVA with treatment (CSF or saline and TRPV1 antagonist or vehicle, respectively) as the independent factor and time as the repeated factor was used for statistical analyses of changes in mechanical head withdrawal thresholds for each side separately (ipsi- and contralateral). Bonferroni was used as post-hoc test when the ANOVA indicated an overall significance. The *t*-test with Welch correction was used to analyze differences between the two groups. A probability level of less than 0.05 was considered for statistical significance.

## Results

### Masseter muscle inflammation induced by CFA

**Figure 1:**
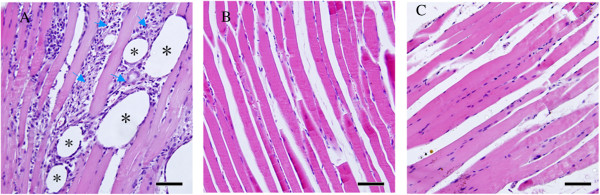
**Morphological indications of inflammation occurred in the masseter muscle following injection of CFA. (A)** Haematoxylin and eosin staining 4 days following injection of CFA in masseter muscle; note the extensive infiltration of inflammatory cells (indicated in arrows) and vacuoles (indicated in asterisks). **(B)** Masseter muscle contralateral to CFA injected side. **(C)** Masseter muscle 4 days after injection of saline, and no signs of inflammation were observed in saline injected and contralateral masseter muscle. The scale bar represents 20 μm.

Unilateral injection of CFA into masseter muscle induced edema on the injected side. This sign of edema appeared 4 hours after injection and persisted for at least 4 days. No edema was observed at contralateral masseter or masseter injected with saline. Histopathological examination showed qualitative differences between muscles injected with CFA and saline. Injection of CFA into the masseter muscle produced clear signs of inflammation characterized by extensive infiltration of granular leukocytes and numerous vacuoles at site of injection (Figure 1A). In contrast, there were no histological signs of inflammation at neither contralateral nor saline injected masseter muscles (Figure 1B,C).

### Bilateral mechanical allodynia was induced after unilateral injection of CFA into masseter muscle

**Figure 2:**
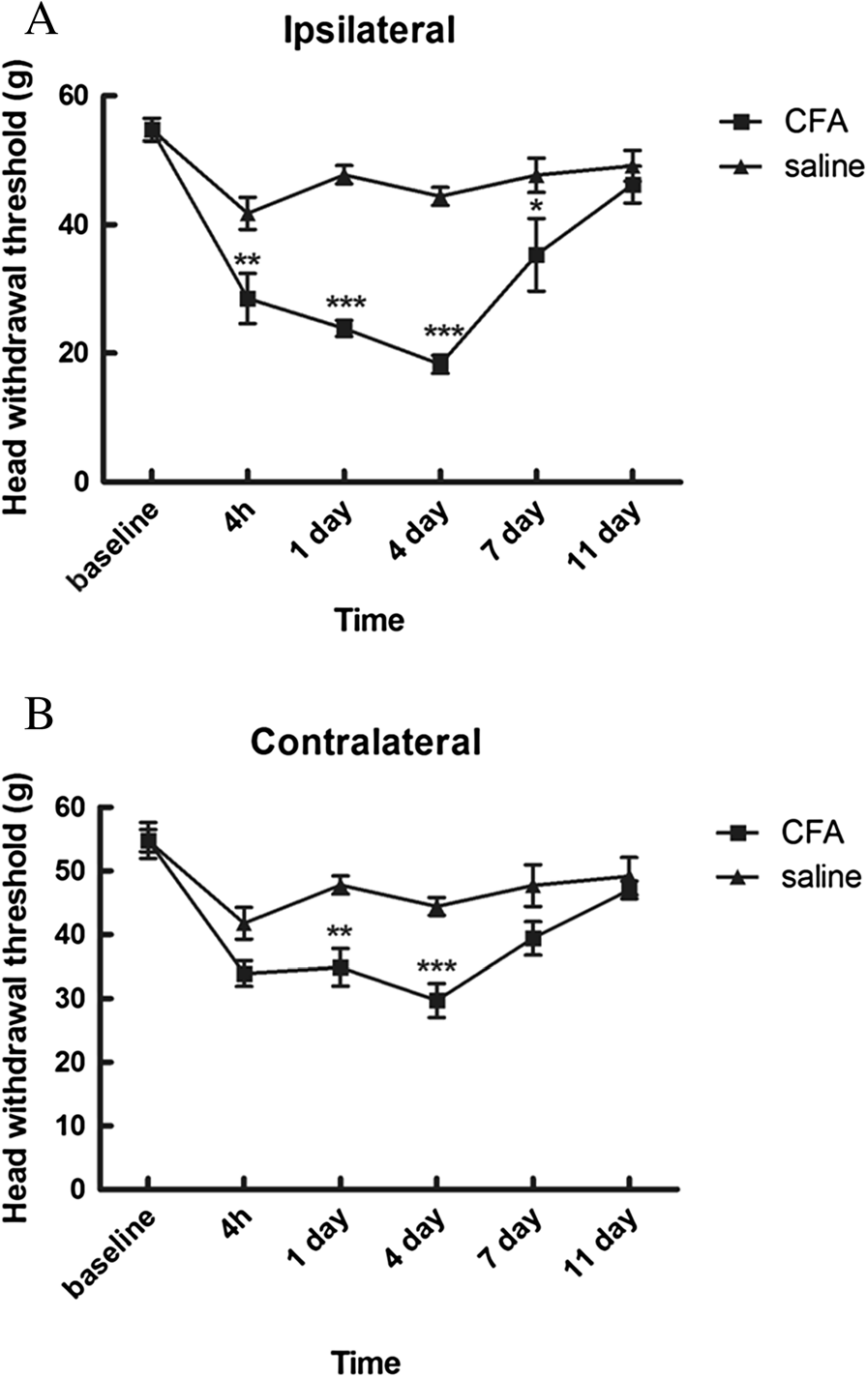
**The time course of head withdrawal thresholds following injection of CFA and saline into the masseter muscle.** Unilateral injection of CFA into the masseter muscle induces allodynia in ipsilateral masseter at 4 h, day 1, day 4 and day 7 after injection. **(A)** Head withdrawal thresholds for the ipsilateral masseter (two-way ANOVA followed by Bonferroni *post-hoc*, n = 6). Allodynia of contralateral masseter was observed at time point of day 1and day 4. **(B)** Contralateral masseter (two-way ANOVA followed by Bonferroni *post hoc*, n = 6). Results are presented as means ± s.e.m. *p < 0.05; ** *p* < 0.01;*** *p* < 0.001 versus saline group at same time point. Note the lowest head withdrawal thresholds at bilateral masseter muscle 4 days after CFA injection.

To examine whether unilateral inflammation of masseter muscle can affect pain response at site of CFA injection and contralateral side, bilateral head withdrawal threshold was measured. Reduction in head withdrawal threshold was found not only in the ipsilateral side but also the contralateral side following a unilateral injection of CFA into the masseter muscle, as compared with saline injected control animals. On the side ipsilateral to CFA injection, the head withdrawal threshold started to decrease at 4 h (*p < 0.01*), reached its peak at day 4 (*p < 0.001)*, and returned to the baseline level at day 11 (*p > 0.05*) (Figure 2A). An increased mechanical sensitivity also developed on the contralateral side (Figure 2B). The decrease in head withdrawal threshold of the contralateral side started at day 1 (*p < 0.01*), also reached the maximum reduction at day 4 (*p < 0.001*), and returned to the baseline level at day 11 after inflammation (Figure 2B). Mechanical hypersensitivity reached the peak on day 4 in both ipsilateral and contralateral masseter muscle, as compared with control animals. These results showed that unilateral CFA injection is capable of inducing bilateral allodynia, although no obvious signs of inflammation were seen in the contralateral masseter muscle.

### Masseter muscle inflammatory allodynia up-regulates TRPV1 expression in the trigeminal ganglion

**Figure 3:**
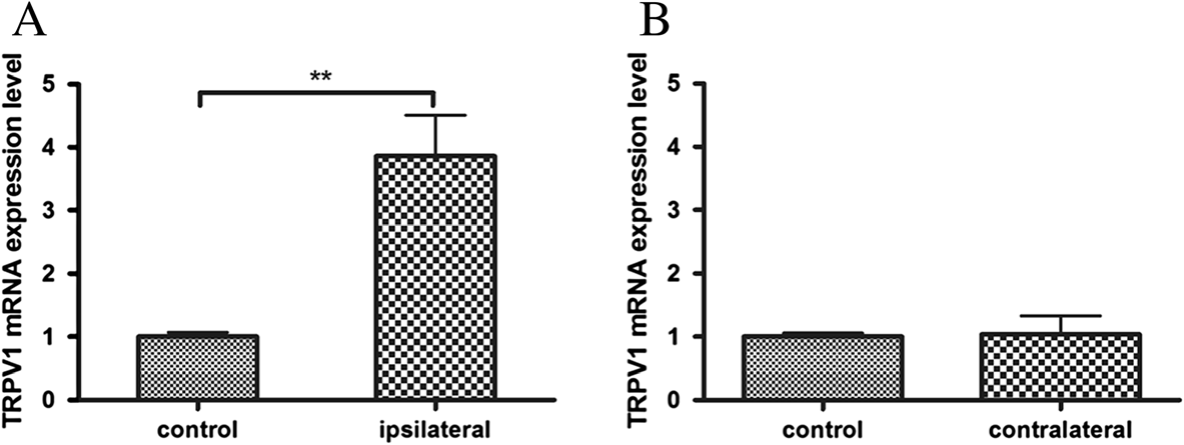
**TRPV1 mRNA expressions in the trigeminal ganglion. (A)** Real-time PCR revealed up-regulation of TRPV1 expression after masseter inflammation in ipsilateral TRG; **(B)** There was no difference in TRPV1 expression in contralateral TRG. Data are presented as means ± s.e.m., n = 8. **p < 0.01 versus control group, using t-test.

Real-time PCR was used to examine changes in TRPV1 mRNA expression level in ipsilateral and contralateral trigeminal ganglion (TRG) after unilateral injection of CFA. TRPV1 mRNA levels were measured at post-injection day 4 when the rats exhibited significant reduction in bilateral head withdrawal threshold (Figure 2A, ipsilateral head *p* < 0.0001; B, contralateral head *p* = 0.0016, as compared with control animals), reconfirming the development of bilateral allodynia after unilateral masseter muscle CFA injection (as shown in Figure 2). Significant increase of TRPV1 mRNA in the ipsilateral TRG was found following CFA injection, as compared with the controls (Figure 3A, top panel; *p* = 0.0031), while there was no alteration in TRPV1 mRNA in the contralateral TRG (Figure 3B bottom panel; *p* = 0.9159). These results show that unilateral injection of CFA into masseter muscle up-regulates TRPV1 expression in ipsilateral TRG, and appears not to affect TRPV1 expression level in contralateral TRG.

### Intrahippocampal injection of TRPV1 antagonist attenuate bilateral masseter muscle allodynia

**Figure 4:**
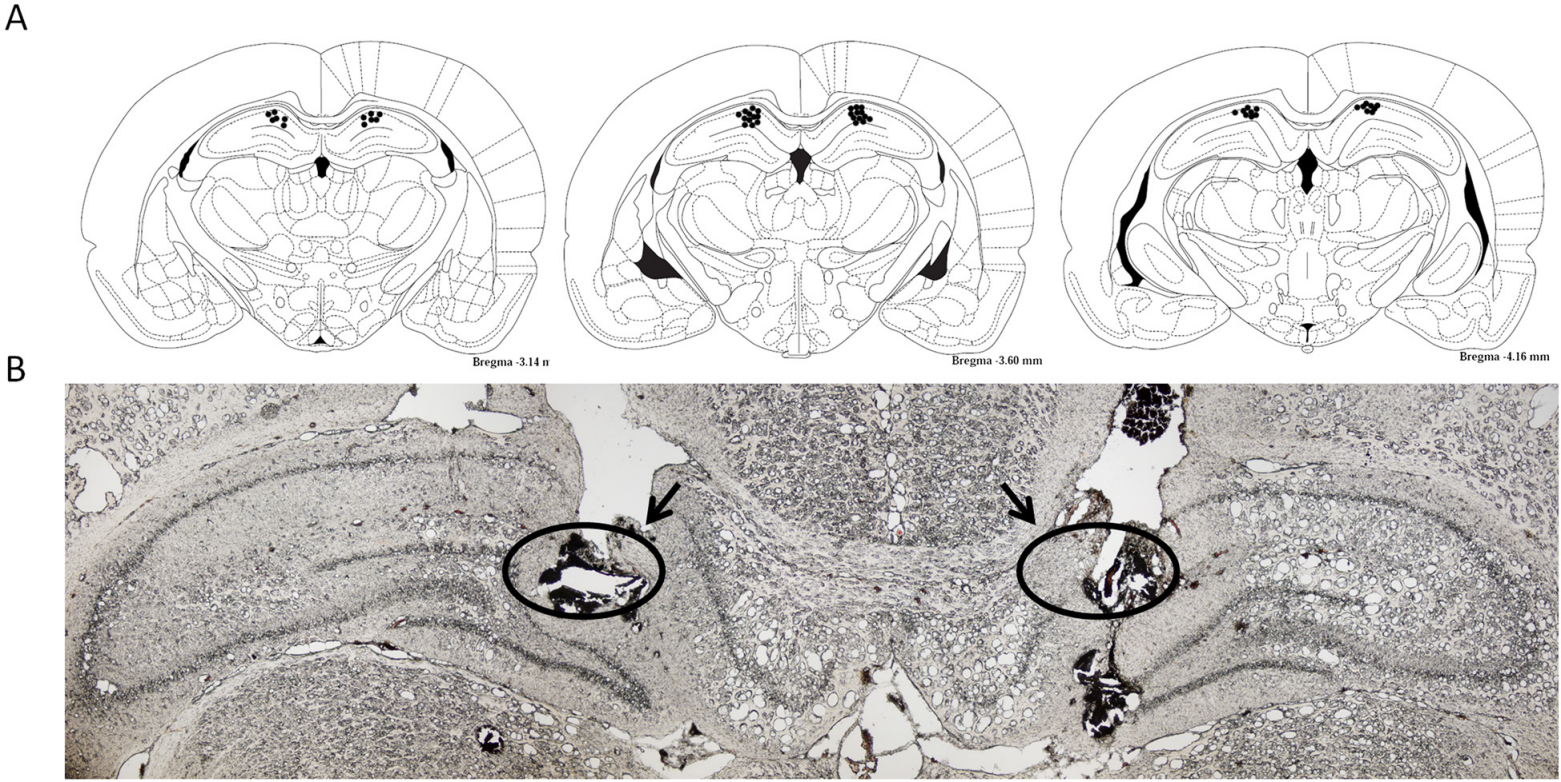
**Confirmation of cannula placement in the hippocampus. (A)** Reconstruction of serial coronal sections of the brain illustrates the bilateral injection sites of the cannulas. The black dots on the schematic illustration of the coronal section of rat brain indicate the placements of cannulas in the brain. **(B)** Representative microphotograph of the coronal section shows the placements (indicated by circles and arrows) of a pair of cannulas in the CA1 region of the hippocampus.

To test whether bilateral nocifensive behavioral response after unilateral masseter muscle inflammation is due to changes in TRPV1 at hippocampal level of the brain, we performed injection of specific TRPV1 antagonist 5-iodo-resiniferatoxin into the CA1 region of hippocampus. By confirmation of placement of cannulas within the CA1 region of hippocampus between bregma −3.14 mm and −4.16 mm, we confirmed that the drugs were successfully injected into the target sites (Figure 4).

**Figure 5:**
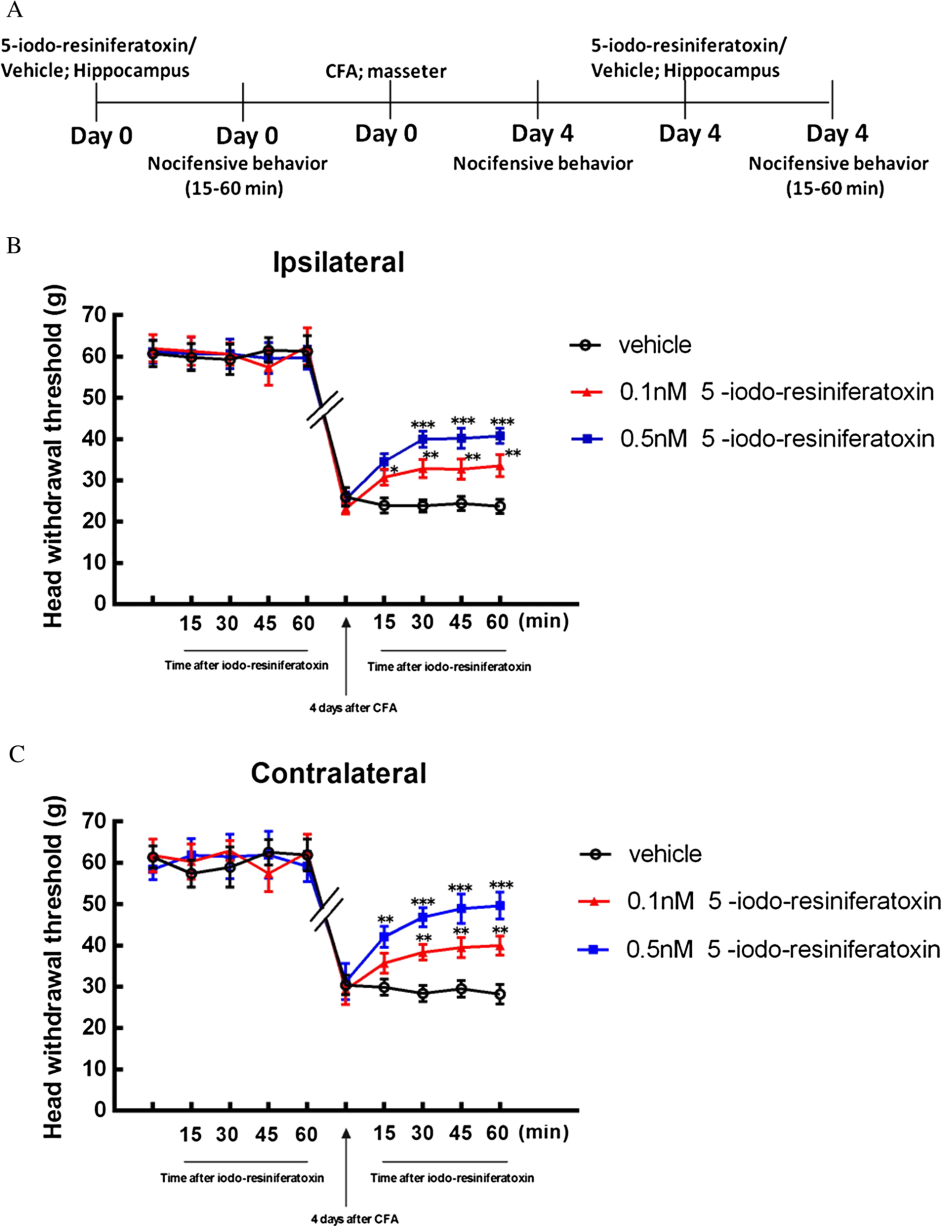
**Attenuation of bilateral masseter muscle allodynia by TRPV1 antagonist.** Experimental design of intrahippocampal injection of TRPV1 antagonists **(A)**. The head withdrawal threshold was not different between groups with intrahippocampal injection of 5-iodo-resiniferatoxin (0.1 or 0.5 nmol) and vehicle injected group before induction of masseter muscle inflammation (*p* > 0.05). Significant decrease in head withdrawal threshold 4 days after CFA injection in both ipsilateral and contralateral masseter confirmed the development of allodynia **(B, C)**. The attenuation of allodynia of ipsilateral masseter was observed 30 min, 45 min and 60 min after intrahippocampal injections of 5-iodo-resiniferatoxin **(B)** and in all time points in contralateral masseter **(C)**. Data are presented as means ± s.e.m., n = 6. * *p* < 0.05; ** *p* < 0.01; *** *p* < 0.001 versus vehicle group at the same time point, using two-way ANOVA followed by Bonferroni *post- hoc.*

Intrahippocampal injection 0.1 or 0.5 nmol of 5-iodoresiferatoxin or vehicle did not affect the baseline of head withdrawal threshold before injection of CFA in masseter muscle (Figure 2 and Figure 5A,B). The bilateral head withdrawal threshold was significantly decreased 4 days after unilateral injection of CFA in masseter muscle, confirming the development of allodynia in bilateral masseters (Figure 5B,C). After intrahippocampal injection of 0.1 or 0.5 nmol 5-iodoresiferatoxin, the attenuation of bilateral allodynia was observed 30, 45 and 60 min in ipsilateral masseter (*p* < 0.01 for group of 0.1 nmol and *p* < 0.001 for group of 0.5 nmol) (Figure 5B) and 15, 30, 45 and 60 min in contralateral masseter (*p* < 0.01 for group of 0.1 nmol and *p* < 0.001 for group of 0.5 nmol) (Figure 5C), with the strongest reversal effect of 0.5 nmol 5-iodoresiferatoxin on withdrawal threshold at 60 min about 73% for ipsilateral and 87% for contralateral, respectively. These results demonstrate that blocking hippocampal TRPV1 can attenuate bilateral allodynia after unilateral injection of CFA in masseter muscle, suggesting that the nociceptive response at side contralateral to CFA induced inflammation is likely mediated through central neural mechanism.

## Discussion

The purpose of this study was to investigate whether unilateral inflammation of masseter muscle can cause a bilateral allodynia in rats and if so, whether TRPV1 channels may contribute to the bilateral allodynia. We took advantage of the CFA models of inflammation that have been used in studies investigating nociception in the orofacial region [16,37,39]. Our findings show the nocifensive behavioral response in both inflamed and contralateral non-inflamed masseter muscle. The mechanism of bilateral nocifensive behavioral response is not fully understood although bilateral allodynia after unilateral inflammation of deep craniofacial tissues has also been shown in recent studies [11,36]. Characteristics of contralateral events after unilateral injury that follows the same pattern as at ipsilateral side but usually smaller in magnitude with briefer time course are well documented [40]. Unilateral injection of CFA in masseter muscle complex is capable of inducing bilateral and also widespread hyperalgesia involving different parts of body in rats [11], and unilateral injection of hypertonic saline in masseter or overlying skin evokes bilateral enhancement of signal intensity in the brain in humans [14].

In this study we found bilateral nocifensive behavioral response 1 day and 4 days after unilateral injection of CFA. This result confirms induction of bilateral allodynia by unilateral deep tissue inflammation in orofacial region. Mechanical hypersensitivity developed almost immediately at site of CFA injection and the highest hypersensitivity reached at day 4. Contralateral behavioral response to mechanical stimuli follows the same pattern as the ipsilateral inflamed side, although not as immediately nor as robustly as the injection side. Head withdrawal threshold reached lowest and highly significant difference in both ipsilateral and contralateral masseter 4 days after CFA injection. Similar to this study in rats, clinical studies report bilateral pain in patients with myofascial temperomandibular disorder [41,42]. Besides bilateral hypersensitivity, overlaps of symptoms especially in hypersensitivity disorders associated with nociceptive impairment like fibromyalgia and whiplash injury suggest that alterations in nociceptive processing may also play role in etiopathogenesis of orofacial pain [43,44].

Previous studies from ours and others have reported the involvement of TRPV1 in orofacial pain, and its importance in muscle nociception and mechanical hyperalgesia in animal models [16,18]. In this study, real time RT PCR was performed to detect alterations in TRPV1 mRNA expression in bilateral TRG after unilateral masseter inflammation. Up-regulation of TRPV1 mRNA expression in the trigeminal ganglion ipsilateral to inflammation found in this study confirms the importance of TRPV1 in inflammatory orofacial pain conditions [24,45]. No changes in expression level of TRPV1 in contralateral TRG suggest that changes in ipsilateral TRG are specific to inflamed tissues. However, it is noteworthy to mention that TRPV1 is expressed not only in neurons but also in mast cells as TRPV1 expression can be induced in fibroblasts following inflammation [46-48]. Therefore, the upregulation TRPV1 observed in this study cannot exclude non-neuronal type of cells in TRG, although the great majority of expression is in neurons. The finding of increased calcitonin gene-related peptide (CGRP) mRNA level just in ipsilateral TRG in animals with bilateral allodynia also supports the view that changes in TRG are specific to inflammation and nerves innervating inflamed tissue [49]. An increased mechanical sensitivity of the contralateral side observed in this and previous studies suggests development of secondary mechanical allodynia after masseter inflammation [11,36]. The role of central mechanisms in contralateral pain response of masseter muscle has previously been suggested by results of bilateral c-fos expression in the brainstem after unilateral inflammation of masseter muscle [50]. Beside TRPV1 are expressed in the sensory nerves of peripheral nervous system, including masseter muscle afferents in the TRG [20], a significant increase in TRPV1 expression is found in hippocampus after TMJ inflammation [16]. Since the effect of TMJ inflammation on TRPV1 expression in the hippocampus is specific but in other brain regions (16), we decided to test if blocking the TRPV1 in CA1 region of hippocampus can attenuate bilateral masseter muscle pain response as well. In this study, the involvement of central mechanism in masseter pain conditions was supported by results of reversed head withdrawal threshold in both ipsilateral and in contralateral masseter after hippocampal injection of TRPV1 antagonist, suggesting that blocking the function of hippocampal TRPV1 can attenuate bilateral masseter muscle allodynia in rats. While ipsilateral nociception seems to depend on alterations in both peripheral and central neurons, contralateral response seems to depend mainly on alterations in higher neural centers. Although it was reasonable to expect changes in nocifensive behavioral response at the inflamed muscle site, the contralateral allodynia suggests that inflammation of masticatory muscles is likely a peripheral trigger of CNS alterations that lead to nociception of both inflamed and non inflamed tissue.

In conclusion, the results of this study support the possibility of bilateral changes in pain response after unilateral inflammation of masseter muscle in rats, and suggest the involvement of TRPV1 channels in masseter muscle pain conditions.

## Competing interests

The authors declare that they have no competing interests.

## Authors' contributions

SSK carried out behavioral assays, animal surgery, histological experiments and drafted the manuscript. XHZ performed behavioral experiments. WL, YWW and IV were responsible for performance of quantitative real-time PCR. KWW participated in the study design and wrote the manuscript. All authors have read and approved the final manuscript.
